# A new three-step hybrid approach is a safe procedure for incisional hernia: early experiences with a single centre retrospective cohort

**DOI:** 10.1007/s10029-020-02300-9

**Published:** 2020-09-12

**Authors:** L. Matthijs van den Dop, Gijs H. J. de Smet, Michaël P. A. Bus, Johan F. Lange, Sascha M. P. Koch, Willem E. Hueting

**Affiliations:** 1 Department of Surgery, Erasmus University Medical Centre, Room Ee-173, Dr. Molewaterplein 40, 3000 CA, PO BOX 2040, 3015 GD Rotterdam, The Netherlands; 2 Department of Surgery, IJsselland Ziekenhuis, Capelle Aan Den IJssel, The Netherlands; 3 Department of Surgery, Alrijne Ziekenhuis, Leiderdop, The Netherlands

**Keywords:** Incisional hernia, Hybrid, Laparoscopic, Surgical technique, Hernia recurrence, Enterotomy

## Abstract

**Purpose:**

In this study, a three-step novel surgical technique was developed for incisional hernia, in which a laparoscopic procedure with a mini-laparotomy is combined: so-called ‘three-step incisional hybrid repair’. The aim of this study was to reduce the risk of intestinal lacerations during adhesiolysis and recurrence rate by better symmetrical overlap placement of the mesh.

**Objectives:**

To evaluate first perioperative outcomes with this technique.

**Methods:**

From 2016 to 2020, 70 patients (65.7% females) with an incisional hernia of > 2 and ≤ 10 cm underwent a elective three-step incisional hybrid repair in two non-academic hospitals performed by two surgeons specialised in abdominal wall surgery. Intra- and postoperative complications, operation time, hospitalisation time and hernia recurrence were assessed.

**Results:**

Mean operation time was 100 min. Mean hernia size was 4.8 cm; 45 patients (64.3%) had a hernia of 1–5 cm, 25 patients (35.7%) of 6–10 cm. Eight patients had a grade 1 complication (11.4%), five patients a grade 2 (7.1%), two patients (2.8%) a grade 4 complication and one patient (1.4%) a grade 5 complication. Five patients had an intraoperative complication (7.0%), two enterotomies, one serosa injury, one omentum bleeding and one laceration of an epigastric vessel. Mean length of stay was 3.3 days. Four patients (5.6%) developed a hernia recurrence during a mean follow-up of 19.5 weeks.

**Conclusion:**

A three-step hybrid incisional hernia repair is a safe alternative for incisional hernia repair. Intraoperative complications rate was low.

## Introduction

Incisional hernias occur in approximately ten to fifteen percent of the general patient population after abdominal surgery [1]. This percentage may exceed over 30% in high risk patients with obesity or an abdominal aorta aneurysm (AAA) [2]. An incisional hernia can develop after any sort of abdominal wall incision. However, abdominal incision through the midline is most prone to incisional hernia development [3, 4].

Known risk factors that contribute to the development of an incisional hernia are high age, high Body Mass Index (BMI), presence of AAA, immunosuppressants, lung disease and heavy physical work. In addition to these risk factors, there are also a number of technical factors that increase the risk of developing an incisional hernia, such as wound infections, abdominal wound dehiscence and suboptimal closing of the fascia [5–7].

The laparoscopic approach is widely used since it was first introduced by LeBlanc et al*.* [8] in 1993 and consists of the placement of the mesh in an intraperitoneal onlay (IPOM) manner. The mesh is fixated to the abdominal wall with the use of tackers or/and sutures [9]. Laparoscopic approach seems to have an advantage over an open approach with respect to surgical site infections, shorter hospital stay and less postoperative pain, although it is also associated with more peroperative intestinal lesions, postoperative seroma formation and bulging as compared to the open procedure [10–12]. Bulging and recurrent hernia are especially more present in case the defect has not been closed and in case of insufficient mesh overlap [13]. Seroma infection can lead to infection, mesh removal and hernia recurrence [9, 14–16]. Furthermore, the laparoscopic approach is associated with the risk of enterotomies, serosa bowel injuries and other intraoperative complications, such as bladder injuries, especially for individuals with complex abdominal adhesions [17]. Bowel injury is a relatively uncommon complication, but raises mortality [18].

Recently, there has been some evidence for the benefit for a hybrid incisional hernia repair (a laparoscopic combined with open approach) in terms of enterotomies via safe adhesiolysis at one month follow-up [5]. However, at 1-year follow-up [19], there was no significant difference in respect to recurrence as compared to the pure laparoscopic approach. In both studies of Ahonen-Siirtola et al*.* [5, 19] they used a two-step hybrid approach starting with a mini-laparotomy.

In the newly developed three-step incisional hybrid repair (TIHR) in two locations of a non-academic teaching hospital in the Netherlands, a three-step procedure was used to tackle the disadvantages of the purely laparoscopic approach (i.e., seroma formation, possible bowel injury and bulging and recurrence) and giving way for more options to form an optimal surgical plan. In this study, the first experiences were assessed with this new TIHR with respect to perioperative outcomes, complications and hernia recurrences.

## Methods

This retrospective observational study was conducted according to the STROBE (Strengthening the Reporting of Observational studies in Epidemiology), STROCSS (Strengthening the Reporting of Cohort Studies in Surgery) statements and the EuraHS Working Group (European Registry of Abdominal Wall Hernias) recommendations [20–22].

### Study design

After obtaining approval of the local ethics committee, all patients who underwent elective TIHR in our non-academic teaching hospital between 2016 and 2020, were retrospectively assessed for perioperative outcomes and were included in the study.

Patients were first seen at the outpatient clinic by two dedicated abdominal wall surgeons who performed the intake and physical examination. An incisional hernia was defined as: ‘a ventral hernia that developed after surgical trauma to the abdominal wall, including recurrences after repair of primary ventral hernias’ as described by Muysoms et al*.* [20]. A computed tomography (CT) scan was performed when additional information about the hernia configuration was needed.

Patients were considered eligible for TIHR if the incisional hernia was ≥ 2 cm ≤ 10 cm, and were not pregnant.

### Data collection

The electronic medical records of each patient were retrospectively reviewed to asses baseline patient characteristics (sex, age, smoking, chronic lung ideas, corticosteroid use, diabetes, BMI, ASA classification) hernia characteristics (hernia size, prior operation, multiple defects, location of hernia and radiological imaging used), surgical characteristics (operation time, length of stay (LOS), mesh-type class), intraoperative complications (bleeding or vessel laceration, enterotomy, serosa injury). Postoperative outcomes including wound infections, fever, postoperative pain, seroma and hernia recurrence were assessed during hospital stay and follow-up appointments.

### Surgical planning and patient positioning

**Fig. 1:**
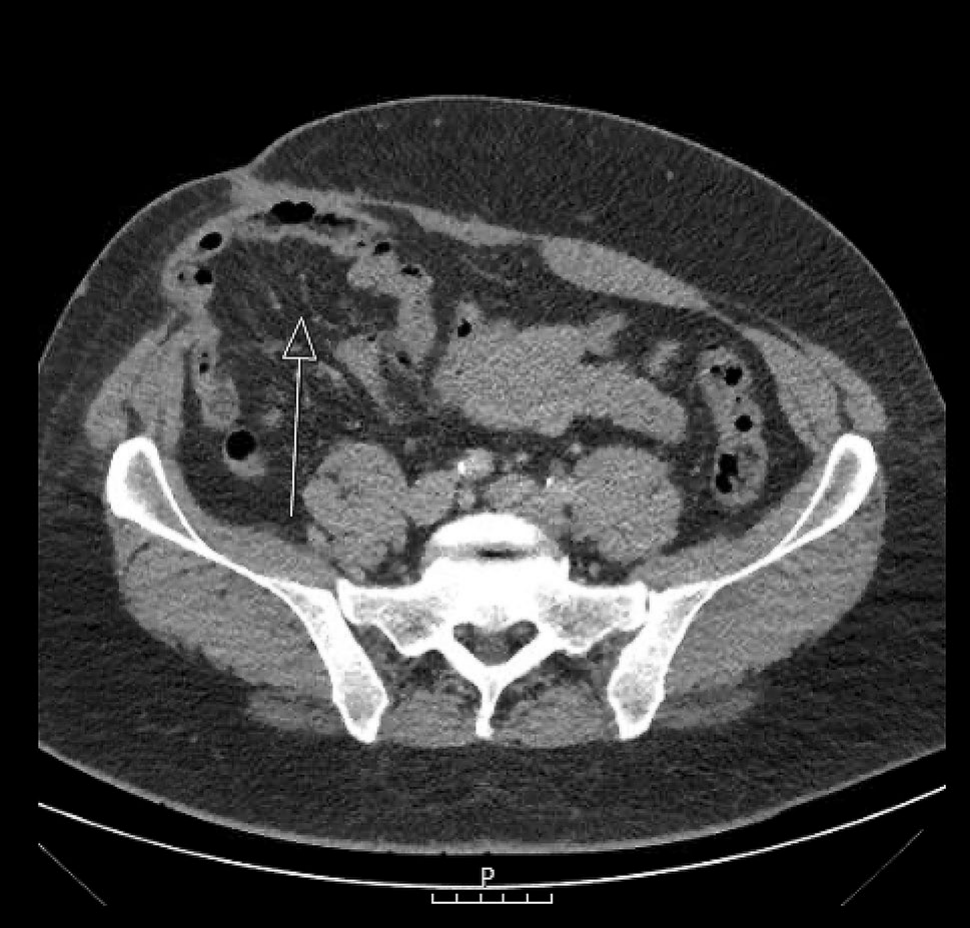
Preoperative CT-scan

The preoperative CT scan helped plan the surgical procedure by providing valuable information about the hernia configuration (Fig. 1). The hernia location, size and contents were documented, as well as the abdominal wall musculature that could be used for mesh implementation.

Surgery was performed under general anaesthesia and antibiotic prophylaxes were administered (cefazolin 2 g, and metronidazole 500 mg if contamination was suspected) in all patients. The patient was positioned in supine position with one arm spread at the side where the hernia is located and the other arm alongside the body. An orogastric tube and Foley catheter were inserted on indication, the operation field was sterilised with chlorhexidine and sterile drapes were placed at all four sides of the patient.

### First laparoscopic part

**Fig. 2:**
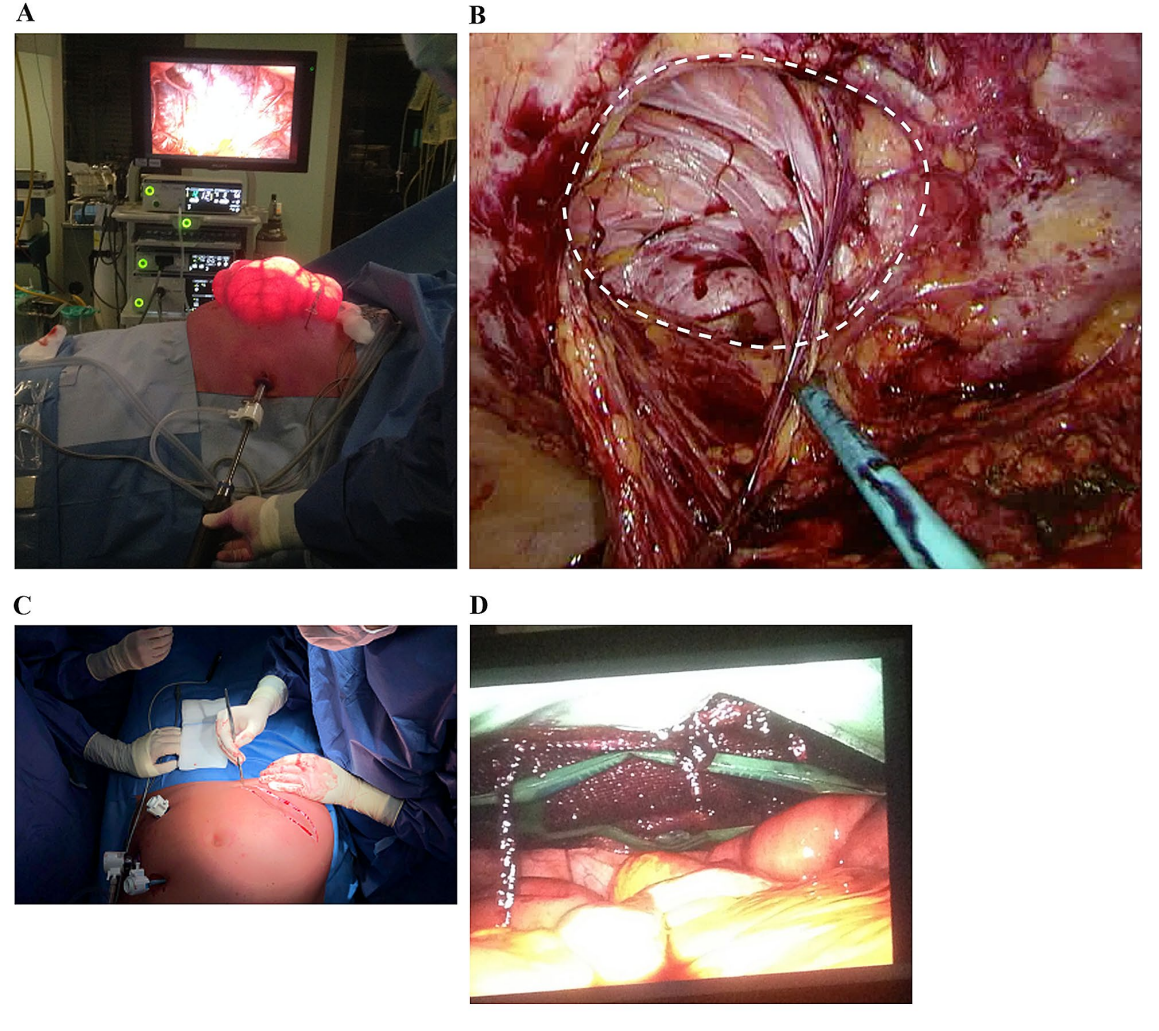
**a** Establishing pneumoperitoneum and placing of the trocars. **b** Laparoscopic adhesiolysis was performed around the abdominal defect for safe continuation of the mini-laparotomy. **c** A mini-laparotomy was performed over the old incision (photo was made with a different patient). **d** The mesh is placed in the centre of the hernia defect so that the mesh surface is spread evenly around the closed defect

First, a “pneumodissection” of the hernia sac was formed with the introduction of a Veress needle in the intra-abdominal space on the left subcostal region. The use of a Veress needle is a standard procedure in the Netherlands and is regarded as acceptable as using the direct trocar insertion technique [23]. This pneumodissection was formed when pressure of the CO_2_ intraperitoneally expands the abdomen and the hernia sac would bulge through the abdominal wall and thereby aiding in determining the length of the laparotomy incision (Fig. 2a). Concomitantly, this would facilitate a safer way for adhesiolysis, gave way for more preservation of abdominal wall, smaller laparotomy incision and optimised the surgical plan for mesh implementation. This preservation of abdominal wall could be advantageous within large hernia defects, because this will give more opportunities for defect closure if the hernia recurs.

A 12 mm visual port was inserted under vision on the contralateral side from the location of the abdominal wall hernia, in case of a midline hernia on de left lateral side, together with two 5 mm trocar ports, both on the contralateral side. The laparoscope was then introduced to inspect the abdominal cavity and identify the hernia sac. Laparoscopic start of adhesiolysis was performed for adhesions around the abdominal defect for safe continuation of the mini-laparotomy (Fig. 2b).

### Second open part

Secondly, the laparoscopic procedure was interrupted, the abdomen deflated and a minimal length excision of the old scar was performed (Fig. 2c). The dissection was continued through the subcutaneous fat and access to the extraperitoneal space was obtained. The hernia sac was opened, open adhesiolysis was performed if laparoscopic adhesiolysis was deemed too dangerous because of unclear view and potential contents of the hernia sac were repositioned in the intra-abdominal space. After excision of the hernia sac, the edges of the fascia were exposed circumferentially. In case of an Echo Positioning System was used, the mesh was put in through the incision and the posterior rectus fascia was closed with PDS (polydiaxone) loop sutures.

### Third laparoscopic part

Thirdly, the procedure was continued laparoscopically, the abdomen inflated and in the case of a Ventralight ST mesh, the mesh was now introduced into the abdominal cavity through the laparoscopic port. With the use of a Sorbafix tacker, the mesh was fixated to the abdominal wall. When using the Echo mesh, a disposable titanium body was fixated on the mesh. Through the expansion of the titanium body inside the abdomen, the mesh could easily be positioned in the centre of the closed hernia defect and ensure that the mesh surface was spread evenly (Fig. 2d). After the fixation in a single or double crown manner, the titanium body was evacuated. In case of use of a Ventralight ST mesh, 4 transfascial sutures with resorbable polyglactin were used for correct positioning before tackering. Lastly, a laparoscopic overview was used to ensure correct mesh position. The trocars and Veress needle were always removed under vision, closed suction drains were placed under the subcutaneous space if the woundsurface deemed considerably of size. The subcutaneous fat was approximated with Vicryl and the skin was sutured with resorbable monofilament sutures.

### Primary and secondary outcomes

The primary outcome was intraoperative complications (i.e., bowel injury, serosa injury or other complications related to the operative procedure). Secondary outcomes were LOS, recurrence and postoperative complications (classified with the Clavien–Dindo classification of surgical complications [24]).

### Follow-up

Patients were seen one month after surgery at the outpatient clinic by the surgeon who performed the surgery. Postoperative discomfort was discussed and physical examination was performed. If patients did not develop new complaints, they were discharged from follow-up. Patients were instructed to contact the hospital if new symptoms emerged.

### Statistical analysis

Statistical analysis was performed with SPSS software version 26. Continuous variables are presented as mean and standard deviation (SD) or median. Discrete variables are presented as absolute numbers and percentages.

## Results

A total of 82 patients that underwent TIHR were retrospectively analysed. Four patients were excluded because they had a two-step hybrid procedure, two patients were excluded because the initially TIHR procedure was converted to an open procedure due to primary closing of a small defect size (1.5 cm) and a defect that had a width of 12 cm, requiring a transverse incision. Two patients had a primary ventral hernia and four patients had a hernia defect that was larger than 10 cm. All patients were operated between December 2016 and March 2020.

### Patient baseline characteristics

The patient baseline characteristics are shown in Table 1. Forty-six patients (65.7%) were females and the mean age was 59 years with a standard deviation of 12 years. Mean follow-up duration was 19.5 weeks (± 25.3).

**Table 1:** Patient baseline characteristics for patients with TIHR

TIHR	*N* = 70 (%)
Sex	
Male	24 (34.3)
Female	46 (65.7)
Age (years)	59 (12.0)
Smokers	14 (20.0)
Chronic lung disease	14 (20.0)
Corticosteroid use	11 (15.7)
Diabetes	11 (15.7)
BMI (kg/m^3^)	30 (6.1)
ASA classification	
1	5 (7.1)
2	44 (62.9)
3	21 (30)
Follow-up (weeks)	19.5 (25.3)

Continuous variables are presented as mean and (SD). Discrete variable are presented as absolute number and (percentage)

*ASA *American society of anaesthesiologist, *BMI* body mass index

### Hernia characteristics

Hernia characteristics are presented in Table 2. Mean hernia size in width was 4.8 (± 2.4) centimetres. The most common surgery subtype prior to the incisional hernia was gastrointestinal surgery with 60% of the incisional hernias. Other common surgical procedures prior to developing an incisional hernia were primary ventral hernia repairs. In 38.6% of all cases the hernia was located in the midline and 25.7% of patients had multiple hernia defects.

**Table 2:** Hernia characteristics for patients with TIHR

Hernia characteristics	*N* = 70 (%)
Hernia size (mean width in cm)	4.8 (2.4)
2–5 cm	45 (64.3)
6–10 cm	25 (35.7)
Prior operations	
Open	43 (61.4)
Laparoscopic	27 (38.6)
Operation subtype	
Gastrointestinal disease	42 (60.0)
Abdominal wall hernia	12 (17.1)
Gynaecological disease	7 (10.0)
Urological disease	6 (8.5)
Vascular disease	3 (4.3)
Location of hernia	
Midline	27 (38.6)
Paramedian	15 (21.4)
Flank	15 (21.4)
Parastomal	5 (7.1)
Trocar	3 (4.3)
Hypogastric	3 (4.3)
Patients with multiple defects	18 (25.7)
Radiology	
CT scan	44 (62.8)
Ultrasound	21 (30.0)
CT and ultrasound	3 (4.3)
No radiology performed	2 (2.9)

Continuous variables are presented a mean and (SD). Discrete variable are presented as absolute number and (percentage)

### Surgical characteristics

Surgical characteristics are presented in Table 3. The mean operation time was 100.4 min with a standard deviation of 44.8 min. LOS was 3.3 days, with a standard deviation of 3.0 days. There were five intraoperative complications (7.0%). One laceration of an epigastric vessel, one serosa injury and two enterotomies of the small bowel that were identified immediately and were sutured with PDS. One patient had a bleeding of the omentum, but this patient was known with severe liver cirrhosis. All patients recovered without further complications. The Ventralight ST mesh was used in most cases (45.7%), next to the Prolene mesh (18.6%). The Echo positioning system was used in 27.2% of cases depending on the surgeon’s preference. The biological Phasix mesh was used when there was doubt about contamination during the procedure (7.1%).

**Table 3:** Surgical characteristics for patients with TIHR

Surgical characteristics	
Operation time (minutes)	100 (44.8)
LOS (days)	3.3 (3.0)
Mesh type class^a^	*N* = 70 (%)
3 (Ventralight ST)	32 (72.9)
2a (Prolene)	13 (18.6)
6b (Phasix)	5 (7.1)
3 (Sepramesh ST)	1 (1.4)

Continuous variables are presented as mean and (SD). Discrete variable are presented as absolute number and (percentage)

*LOS* length of stay

^a^ Classification system used as described by Klinge et al. [41]

### Postoperative outcomes

Postoperative complications are shown in Table 4. The most common postoperative complication was postoperative pain, that needed additional analgesia (5.7%). Postoperative pain medication was paracetamol and nonsteroidal anti-inflammatory drugs (NSAID). This additional analgesia (i.e., opioids) was given after discharge of the patient for one week. There were no patients who developed chronic postoperative pain (pain lasting longer than 2 months after surgery with exclusion for other causes as defined by Macrae et al*. *[25]). One patient had low haemoglobin levels postoperatively, which required blood transfusion. Two patients developed hypotension, which had to be managed at the ICU for one night. Both patients recovered after administration of antiarrhythmic medication. Two patients suffered from respirator insufficiency, with one patient having an unknown giant hiatal hernia with multiple organs in the chest. Another patient had a community acquired pneumonia who had to be transfered to the ICU because of low saturation. One patient known with severe comorbidities developed abdominal sepsis after the operation and deceased after 15 days. Four patients (5.6%) developed a hernia recurrence during a mean follow-up time of 19.5 weeks and one patient was lost during follow-up.

**Table 4:** Complications in patients with TIHR

Complications	*N* = 70 (%)	Clavien-Dindo
Intraoperative complications	5 (7.0)	
Laceration of a. or v. epigastrica	1 (1.4)	
Enterotomy of small bowel	2 (2.8)	
Serosa injury	1 (1.4)	
Bleeding of omentum	1 (1.4)	
Postoperative complications	18 (25.7)	
Hospital acquired pneumonia	2 (2.8)	2
Hypotension	2 (2.8)	2
Seroma	2 (2.8)	1
Surgical site infection	1 (1.4)	1
Gastroparesis	1 (1.4)	1
Low haemoglobin	1 (1.4)	2
Respirator insufficiency	2 (2.8)	4
Problems with intake	1 (1.4)	1
Prolonged pain requiring analgesia	4 (5.6)	1
Haematoma	1 (1.4)	1
Postoperative abdominal sepsis	1 (1.4)	5
Hernia recurrence	4 (5.6)	

Discrete variables are presented as absolute number and (percentage), Clavien–Dindo classification is used for the classification of surgical postoperative complications

## Discussion

Incisional hernia repair remains a challenging issue. In this study, a novel technique was used and evaluated based on the perioperative outcomes. The incidence of intraoperative complications was low (7.0%) and most postoperative complications did not seem to have a relationship with the procedure itself. Therefore, the three-step TIHR appears to be a safe procedure.

Using the three-step TIHR procedure, adhesions around and within the abdominal wall defect and intestines can be visualised more clearly after the pneumodissection is established. Subsequently, the surgeon is able to safely perform the adhesiolysis laparoscopically before entering the abdominal space via the mini-laparotomy. In doing so, the risk of enterotomy is minimalised and the surgeon is able to give way for a patient tailored hernia procedure.

Another advantage of this three-step TIHR is the preservation of abdominal wall. Through foresight of the hernia configuration, optimal excision of the hernia sac could be achieved when desufflating the pneumoperitoneum. By this optimal excision, preservation of abdominal wall tissue could be facilitated and therefore, giving more options for further surgery if the patients develops a recurrence.

Other studies reporting on ventral hernia hybrid repair procedures appear to be promising as well [5, 19, 26–33]. However, these studies included a low number of patients and multiple types of different hernias (primary, incisional, flank or stomal). Ahonen-Siirtola et al*.* [5, 19] was the first who performed a randomised control trial (RCT) on hybrid incisional hernia repair and found promising results for intraoperative complications as compared to laparoscopic incisional hernia repair (LIHR) with respect to incidence of enterotomies (1.1% *vs* 5.3%).

Other hybrid procedure studies reported intraoperative complications ranging from 0 to 16.7% [5, 28–30, 33]. In the study that reported 16.7% intraoperative complications, they only treated patients with complex abdominal adhesions [23]. Despite the small sample size of these studies, a positive trend is visible in respect to hybrid procedures. The intraoperative complication rate of LIHR procedures is reported to be 9.6–13.2% [17, 28, 34]. Moreover, the enterotomies reported by Ahonen-Siirtola et al*.* [28] were all detected during the hybrid procedure, while 4 of the 5 bowel injuries in the laparoscopic group were undetected at the time of surgery, and one of those patients died after developing a septic shock as a consequence of the bowel injury 69 days later.

Other hypothesised advantages of the hybrid repair were a lower rate of seroma formation and bulging when closing the preperitoneal fascial defect. A study by Zeichen et al*.* [35] comparing closing of the preperitoneal defect with subsequent laparoscopic ventral hernia repair (IPOM-Plus) *versus* laparoscopic ventral hernia repair with normal IPOM showed significant reduction in the incidence of seroma formation. Another study of Clapp et al*.* [36] found a significant reduction in bulging after using IPOM-Plus in LVHR. However, in a RCT by Lambrechts et al*.* [37] these two aspects were similar in both groups (IPOM-Plus vs*.* standard IPOM). In the meta-analysis of Awaiz et al*.* [10] on the subject of elective LIHR versus open repair, seroma incidence in LIHR has been reported to be 4.4–35.5%. In the RCT of Ahonen-Siirtola et al. [5] the clinically observed seroma formation was 48.9% in the laparoscopic group and 31.4% in the hybrid group. In this study, a fairly low seroma incidence was reported (2.8%). This could be due to the fact that patients were only seen one time postoperatively at the outpatient clinic without radiological check-up and only returned when they reported complaints, underestimating the seroma incidence.

Postoperative bulging is recognised as an adverse outcome in LVHR and can be perceived as cosmetically dissatisfying. The anatomic aetiology of bulging is that neither the hernia defect nor the rectus diastasis was closed during the hernia repair [38]. It is to the belief of the IEHS (International EndoHernia Society) that ‘a failure to position/fix the mesh flat may contribute to postoperative bulging’ and they recommend that ‘the mesh should be tensioned appropriately such that the mesh is flat without any wrinkles/folds following desufflation of the abdomen’. In this study, by ensuring an even underlay with the third-step as laparoscopic overview, a flat mesh fixation is attempted, also reducing the risk of enterocutaneous fistula formation by wrinkling of the mesh.

Postoperative complications occurred in 25.7% of the patients in this study. This complication rate is comparable with studies involving laparoscopic incisional hernia repair [34, 39–41]. A reason for this complication rate could be that the 32% of patients had an ASA classification of 3. These patients were more prone to develop postoperative complications due to their comorbidities.

Recurrences in hybrid incisional hernia repairs are somewhat underreported due to the novelty of the procedure and studies. In the RCT by Ahonen-Siirtola et al. [19], at 1-year follow-up, no statistically significant difference in hernia recurrence rates between both procedures was found (LIHR 7% *vs* hybrid incisional hernia repair 6%). Other hybrid incisional hernia repairs studies have a very low number of patients included in the studies, which makes it hard to draw conclusion about recurrence rates [27, 29, 30]. Recurrence rates found in LIHR are reported to be 9.7% (12 months)—24.0% (35 months) [17, 40]. The recurrence rate in this study was 5.6% with a mean follow-up time of 19.5 weeks. Owing to the short follow-up period, no hard conclusion could be drawn from this recurrence rate.

Two dedicated abdominal wall surgeons performed the procedure. WH was the first surgeon to incorporate this procedure in patients with incisional hernias. To perform hybrid procedures for SK as well, an one-time participation in a hybrid procedure with WH was needed to attain the skills needed for implementing this procedure in the daily practice of SK. Therefore, it could be stated that this procedure is well feasible to transfer amongst abdominal wall surgeons with laparoscopic expertise, without raising costs or extending a large amount of operation time.

## Limitations

This study has some limitations. First, the outcomes of this study are not based on a randomised data and all data were retrospectively collected and analysed, which gives a potential risk of selection bias. Furthermore, the follow-up appointments were only made one month pos. After this appointment, patients were instructed to return to the outpatient clinic if new complaints occurred. This shortened the follow-up duration and may have underestimated long-term postoperative complications, such as hernia recurrences, as well as seromas. On the other hand, patients were clearly instructed with signs and symptoms and when to reach out to the outpatient clinic.

## Conclusion

The hybrid approach in case of incisional hernia with a three-step procedure as used in this study is a feasible, safe and easy to incorporate surgical approach. The intraoperative complication rate was low and postoperative complications did not seem to be related to the surgical procedure. Prospective research with standardised radiological follow-up is needed to further affirm the postulated benefits in this study [42].

## Data Availability

All data and material are stored on a computer in the local hospital.

## Code availability

SPSS version 26.0 has been used to do all calculations in this article.
